# Making Clear the Value of Basic Behavioral Research. Commentary: A Crisis in Comparative Psychology: Where Have All the Undergraduates Gone?

**DOI:** 10.3389/fpsyg.2015.01766

**Published:** 2015-11-18

**Authors:** Michael Colombo, Damian Scarf

**Affiliations:** Department of Psychology, University of OtagoDunedin, New Zealand

**Keywords:** comparative psychology, student recruitment, undergraduate courses, graduate programs, education, funding

We agree with Abramson (2015) that the field of comparative psychology faces a problem with respect to student recruitment. We would argue, however, that the problem is much broader and encompasses all domains of *basic behavioral research*, of which comparative psychology is just one. Why is this? Abramson (2015) suggests two primary drivers are the lack of undergraduate courses and the lack of graduate programs on comparative psychology. We would add to this a lack of funding. In fact, based on the current funding climate, it is possible that the few undergraduate and graduate programs that exist will disappear. Why is the funding drying up? One need look not too long ago to the furore created by Congress who challenged the validity of the National Institute of Health's (NIH) peer-review process by singling out a peer-reviewed grant by Professor Edward Wasserman on pigeon's categorization abilities. According to a press release by the office of Rep. Randy Neugebauer (R-Texas), who introduced an amendment that would effectively by-pass NIH's peer-review process, “NIH has continually failed to give a high priority to research on serious mental illnesses.” That may have been true, but obviously Neugebauer felt he would get more traction for his cause by singling out behavioral work done with pigeons for ridicule. And that strategy is borne out of a failure on our part to explain why basic behavioral research is so important.

As Abramson (2015) notes, to improve recruitment, we must make the connection between comparative psychology and human behavior clear. We would add that we must make it clear to students and funding agencies that basic behavioral research lies at the core of every study that seeks to understand human behavior, whether comparative in nature or not. Indeed, behavioral researchers are responsible for the majority of paradigms neuroscientists utilize to study the neural mechanisms of learning and memory. Further, a foundation in behavioral research places students in a strong position to tackle any number of topics in the future. Both authors of this commentary received their PhD training in basic behavioral (animal cognition) research. We both continue to conduct animal cognition studies, but one of us (MC) has embarked on a neuroscience career while the other (DS) has gone on to tackle topics from moral nativism (Scarf et al., 2012a,b) to alcohol consumption in university students (Riordan et al., 2015a,b).

Don't get us wrong. We don't believe that basic behavioral research should exist to merely serve neuroscience, or that students should simply use their behavioral training as a springboard into other domains. Most (hopefully all) of us that conduct basic behavioral research do so because we find understanding human and nonhuman animal behavior fascinating. But as Neugebauer's comments foretell, the argument that understanding animal behavior is fascinating in its own right is becoming a harder and harder point to sell. The nuanced and elegant study conducted by Frank and Wasserman (2005) that finally revealed the equivalence relation of symmetry in nonhuman animals by using a successive go/no-go procedure to control for both spatial and temporal response topographies is nothing short of brilliant, but it will go unappreciated by most outside the field of basic behavioral research. Yet because of this study we are now one step closer to understanding the neural basis of equivalence relations, which underlie many of our complex behaviors (Sidman, 1994).

In conclusion, it is very obvious to us why we need basic behavioral research. But we need to do a far better job of explaining to those outside our field and funding agencies why basic behavioral research is essential. Most importantly, it is critical that we present a unified front.

## Author contributions

MC wrote the first draft. DS revised the manuscript and, along with MC, worked the manuscript into its final format.

### Conflict of interest statement

The authors declare that the research was conducted in the absence of any commercial or financial relationships that could be construed as a potential conflict of interest.
